# Collective modes in three-dimensional magnonic vortex crystals

**DOI:** 10.1038/srep22402

**Published:** 2016-03-02

**Authors:** Max Hänze, Christian F. Adolff, Benedikt Schulte, Jan Möller, Markus Weigand, Guido Meier

**Affiliations:** 1 Institut für Angewandte Physik und Zentrum für Mikrostrukturforschung, Universität Hamburg, 20355 Hamburg, Germany; 2 Max-Planck Institute for Intelligent Systems, Heisenbergstr. 3, 70569 Stuttgart, Germany; 3 Max-Planck Institute for the Structure and Dynamics of Matter, Luruper Chaussee 149, 22761 Hamburg, Germany; 4 The Hamburg Centre for Ultrafast Imaging, Luruper Chaussee 149, 22761 Hamburg, Germany

## Abstract

Collective modes in three-dimensional crystals of stacked permalloy disks with magnetic vortices are investigated by ferromagnetic resonance spectroscopy and scanning transmission X-ray microscopy. The size of the arrangements is increased step by step to identify the different contributions to the interaction between the vortices. These contributions are the key requirement to understand complex dynamics of three dimensional vortex crystals. Both vertical and horizontal coupling determine the collective modes. In-plane dipoles strongly influence the interaction between the disks in the stacks and lead to polarity-dependent resonance frequencies. Weaker contributions discern arrangements with different polarities and circularities that result from the lateral coupling of the stacks and the interaction of the core regions inside a stack. All three contributions are identified in the experiments and are explained in a rigid particle model.

The properties of a crystalline solid are based on the periodicity of its underlying Bravais lattice and the symmetry of its basis. In contrast to ion lattices that naturally affect the behaviour of electrons in solids, metamaterials feature periodic structures that are tailored at will to influence the propagation of the respective excitations^1^, such as electromagnetic radiation in photonic crystals^2^. Magnonic crystals allow controlling the propagation of spin waves^3^. The required periodicity can be found in skyrmion lattices that emerge in unstructured ferromagnetic films^4^. Using nanoscale lithography the periodicity can also be created at will for any other ferromagnetic element. We investigate gyrational waves in coupled magnetic vortices in a tetragonal arrangement^5^,^6^. Although the investigated crystals have a confined size, the fundamental interactions equally apply to larger crystals. This leads to a dispersion relation of gyrational waves that has been observed experimentally in two-dimensional vortex arrangements^7^. Magnetic vortices form in thin film ferromagnetic disks. Their magnetization curls in the plane around the centre where it points out-of-plane^8^. The sense of the in-plane curling of the magnetization defines the circularity (*c* = ±1) of the vortex. The out-of-plane component points either up or down. It defines the polarization (*p* = ±1) and distinguishes the sense of rotation of the gyrotropic motion, the fundamental excitation of the vortex in the sub-Gigahertz range. Since neighbouring vortices couple due to stray fields emerging at the surfaces of the ferromagnetic disks^9^, the motions of closely packed vortices are strongly influenced by surrounding disks^10^,^11^. The interaction between laterally arranged elements has been studied for pairs^12^,^13^, chains^14^,^15^, and two-dimensional arrangements^7^,^16^,^17^. Introducing the third dimension to the vortex lattice allows for a strongly increased packing density and has thus stimulated a variety of studies^18^,^19^,^20^,^21^,^22^,^23^.

Here, we study three-dimensional magnonic vortex crystals by means of ferromagnetic resonance spectroscopy and scanning transmission X-ray microscopy. We investigate the gyrotropic motions of three different arrangements of vortex stacks in dependence on the polarizations and circularities of the individual vortices. The polarization pattern is controlled by self-organized state formation^24^,^25^,^26^. The influence of the vertical and lateral coupling is investigated by comparing different arrangements to calculations within the Thiele model. In lateral arrangements the resonances are determined by the polarities of the vortices only^9^. Due to the interaction of the vortex cores in the stacks the resonance frequencies additionally depend on the circularity. Their origin can be understood quantitatively by coupled out-of-plane dipoles of the disks’ core regions. The application of static magnetic fields results in a separation of the vortex cores and allows for the reduction of the core interaction.

Figure 1 depicts the investigated stacks of polycrystalline permalloy (Ni_80_Fe_20_) disks that are prepared with electron-beam lithography, *in-situ* thermal evaporation of permalloy and silicon layers, and lift-off-processing. Three different types of arrangements have been investigated. They are shown in Fig. 1(a). The arrangements consist of stacks with two (type 1) and three (type 2) disks, as well as 3 × 3 × 3 vortex crystals (type 3). The disks have a diameter of 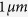 and a thickness of 40 nm for type 1 and 30 nm for type 2 and 3. The silicon spacer has a thickness of 20 nm for type 1 and 30 nm for type 2 and 3. An ensemble of about 100 stacks is deposited on a coplanar waveguide with a thickness of 60 nm of copper and a hydrogen silsesquioxane (HSQ) isolation layer of 30 nm. A sinusoidal current is driven through the signal line of the coplanar waveguide leading to an alternating magnetic field with an amplitude of tenths of milliteslas acting in the plane of the ferromagnetic elements. Scanning transmission X-ray microscopy is performed to spatially resolve the magnetization’s in-plane component. The sample is tilted by 60° degrees with respect to the beam axis. The X-ray measurements are performed at the MAXYMUS microscope of the synchrotron BESSY II in Berlin, Germany. The microscope yields a temporal resolution of 40 ps and a spatial resolution of 25 nm. Magnetic contrast is due to the X-ray magnetic circular dichroism at the nickel 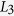 edge at 853 eV. The different possible circularity combinations are encoded in the contrast observed in the micrographs. The transmission through the stacks yields the sum of the in-plane components of the individual layers. Thus, for some cases the circularities in each layer cannot be uniquely identified. Figure 1(b) shows all possible circularity combinations that are matched to X-ray measurements. The silicon interlayer prevents exchange coupling and causes randomly distributed circularities. The polarizations in the stacks are identified by their resonance behaviour as described in the following.

By the unidirectional radio-frequency magnetic field the vortices are excited to rotations around their equilibrium positions. These gyrotropic motions cause the vortex cores to rotate around the centre positions of the disks. The sense of each gyration solely depends on the polarization of the vortex core. Figure 2(a) schematically depicts the relative motions in a stack of two vortices. The deflection of the magnetic vortices leads to a behaviour comparable to coupled in-plane dipoles. Due to the different relative rotations of the two polarity configurations, the in-plane stray-field coupling differs. This yields a splitting of the resonance frequencies as shown in the spectra in Fig. 2(b). A similar behaviour has been observed in systems of two laterally coupled disks^26^. For stacks the splitting of the two polarity states is strongly increased due to the proximity of the disks. Experimentally, the two different relative polarization orientations can be controlled by self-organized state formation^24^. By resonant excitation with a strong amplitude, a polarization pattern can be destroyed. The vortices settle in a stable non-resonant pattern. The state-formation causes all stacks in the ensemble to settle in a mixture of non-resonant states. Two slightly split peaks are observed for the case of equal polarizations. The splitting results from different resonances of stacks with equal and opposite relative circularities in the ensemble. It can be understood within the Thiele model presented in the next paragraph. Figure 2(c) depicts similar resonance spectra for the trilayer stack. Here, we observe three different peaks that can be eliminated from the spectra by a specific state-formation frequency (150 MHz/red, 300 MHz/blue, 450 MHz/green). Each of the three peaks indicated by the black vertical lines in Fig. 2(c) is present in all but one spectrum. Thus, the three peaks can be attributed to the three different relative polarizations indicated by the pictograms. The applied state-formation selectively destroys one state. In contrast to isolated stacks of sample types 1 and 2, crystals of type 3 (Fig. 2(d)) feature an additional lateral coupling between the stacks. Still, the absorption spectra are comparable to those of the isolated stacks. The resonance peaks exhibit an increased line width and are slightly shifted with respect to the ones of the isolated stacks. Supplementary Movie 1 depicts the resonant excitation of a vortex crystal for two different polarization patterns that are individually excited at their resonance frequencies. The two patterns correspond to the red and the green spectrum in Fig. 2(c). The crystal’s vortices in the movie show strong gyrations at their resonance frequency. By interchanging the two excitation frequencies (195 MHz and 413 MHz) the respective pattern is excited off resonance. The large difference of the two resonance frequencies demonstrates the possibilities to tune and manipulate dynamics of three-dimensional vortex crystals. Analytical and numerical calculations elucidate the origin of the observed dynamics. They are presented in the following.

We performed calculations based on the Thiele model^27^ that considers the magnetic vortex as a rigid particle. For the magnetic vortex the Thiele equation reads


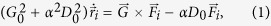


where 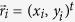 is the relative position of the vortex *i* with respect to the centre of the disk, 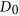 is the dissipation constant, *α* is the Gilbert damping parameter, 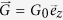 is the gyrovector^28^, and 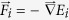 is the force derived from different energy contributions 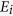. The energy can be separated into three main contributions 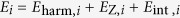 that are described in the following. The confining potential of the permalloy disks is modelled by a two-dimensional harmonic potential 
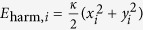
 with a curvature *κ*^28^. The Zeeman energy resulting from an external magnetic field is given by 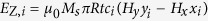 with the saturation magnetization 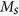, the disk radius *R*, the disk thickness *t*, and the magnetic field 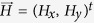. The main contribution to the interaction energy 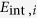 is the energy 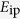 caused by in-plane dipoles. It results from the deflection of the vortex. Here, the interaction between neighbouring vortices *j* and *k* is described via the magnetostatic energy between their side surfaces 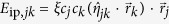 with a scaling factor 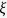 and an interaction matrix 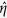 depending on the geometry, the saturation magnetization, and the deflection of the vortices^9^. The scaling factor 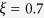 takes into account the theoretically predicted and experimentally observed fact that the coupling is overestimated by the rigid-vortex model^29^,^30^. The inversion of the sign of the surface charges by changing the circularity of the vortex is canceled out by a phase shift of 180° of the excited vortex gyration. The polarity determines the sense of rotation of the in-plane dipoles. Thereby it is solely responsible for different resonance frequencies in laterally coupled disks. This degeneracy vanishes when considering the interaction of the vortex core regions due to the proximity of the cores in the stacks. The interaction is modelled by two magnetic dipoles pointing out-of-plane and can be expressed as a function of the distance of the vortex cores 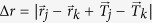, where 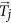 and 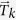 are the centre positions of the disks. The amplitude of the dipole moment is derived from the radius 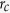 of the vortex core. We assume that all magnetic moments in a cylinder with the radius 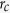 and the thickness *t* point into the out-of-plane direction. The energy of the interaction of the vortex cores is


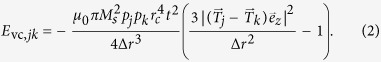


The total energy due to the interaction of the vortices then results in 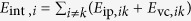. Note that for lateral pairs the contribution of the vortex cores 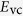 can be neglected. The core interaction is a non-linear contribution that is repulsive for opposite polarizations 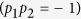 and attractive for equal polarizations 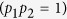. Because the Zeeman energy 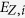 is proportional to the circulation 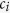 but the contribution of the vortex cores is not, we can already explain the splitting in the absorption spectra as can be seen in Fig. 2(b) for the case of equal polarizations. A stack of two vortices has 4^2^ = 16 and a stack of three vortices has 4^3^ = 64 possible circularity and polarity combinations. Regarding the resonance behaviour we can deduce a reduced number of non-degenerate states from the dependence on the relative polarizations 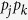 and circularities 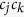 as depicted in Fig. 3(a). All states that comply with the inversion of the polarity or circularity and the mirroring at the symmetry plane of the stack have been grouped into one colour coded state.

The calculations are performed for the experimental spatial sample dimensions. We calculate the steady-state motions for different frequencies of the exciting magnetic field numerically. In contrast to time consuming micromagnetic simulations the absorption spectra are accessible for a high sampling rate of different frequency points, as well as a large number of different circularity and polarity states. The saturation magnetization *M*_*s*_ = 800 kA/m of permalloy is assumed. Due to the different thicknesses of the structures two different resonance frequencies of *ω*_0_/(2*π*) = 240 MHz for elements of type 1 and *ω*_0_/(2*π*) = 210 MHz for elements of type 2 and 3 and a damping coefficient^31^ 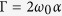 are used. We take a reasonable value of the core radius 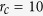 nm^8^ and a Gilbert damping of *α* = 0.01. The magnetic field is given by 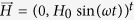, where 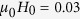 mT. Note that the amplitude of the excitation slightly influences the resonance frequencies due to the non-linearity of the core interaction. This influence is investigated in the last part of this work. The calculations reproduce the spectra obtained from an ensemble of vortices. The different states for the two different stacks and the crystal are calculated individually. The effect of the ensemble is obtained by averaging over the spectra. Figure 3(b) depicts calculations with in-plane coupling 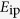 only and with additional core coupling 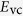 for a stack of two vortices. For the first case there are only two non-degenerate states. In agreement with the measurements we observe an additional splitting of the peaks when the core interaction is taken into account. The frequency and the amplitude of the peaks are in excellent agreement with the measurements. Figure 3(c) depicts the corresponding calculations for the isolated stack consisting of three vortices. They precisely describe the measurements as the calculated splitting can be directly linked to the increased peak widths in the experiments.

In contrast to sample types 1 and 2, samples of type 3 feature an additional coupling of the neighbouring stacks. In a three-dimensional crystal the dynamics depends on the polarizations and, due to the core interactions, on the circularities of all neighbouring vortices. In principle a 3 × 3 × 3 vortex crystal has 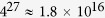 different circularity and polarity combinations. Although some of them are degenerate, it is impractical to calculate all of them individually. To get insight into the lateral coupling mechanism we omit the contribution of the vortex cores. This results in a degeneracy with respect to the circularities leaving 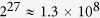 possible polarization states. By the assumption that all nine stacks have the same polarity pattern that can only be inverted with respect to the neighbouring stacks the amount of possible states is reduced to 2^9^ = 512. We calculate the resonance spectra of all 512 combinations in the crystal. This results in an ensemble of resonances that is depicted in Fig. 3(d). Due to the lateral polarization patterns different modes can be excited that vary in their resonance frequencies. This behaviour has also been observed for two-dimensional vortex crystals^7^,^17^. The gyrational modes of the three-dimensional crystal are distributed around the original resonance frequencies of the isolated stack. The average of the ensemble shows a shift towards lower frequencies for the case of equal polarizations in the stacks. This shift matches the observed broadening towards lower frequencies in the experimental spectra (Fig. 2). The lateral coupling is in the same order of magnitude as the coupling of the core regions. To further distinguish between the two mechanisms we took additional spectra that demonstrate the non-linear effect of the interaction of the vortex cores.

For equal polarizations in the stacks Fig. 4 shows absorption spectra obtained for sample type 1 and type 2. We determine the resonances with and without an additional static magnetic field in the plane of the ferromagnetic elements. Lorentzian curves with a constant line width of 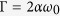 are fitted to the data^31^. The number of fitted peaks originates from the number of non-degenerate circularity combinations. For both sample types we observe a sharpening of the peaks in external magnetic fields. This is due to an increased degeneracy of the circularity configurations when the external magnetic field is applied and can be explained qualitatively. The motions of the vortex cores exhibit a phase shift of 180° for the case of opposite circularities in a pair of stacked disks. This phase shift leads to an attractive force between the core dipoles. The resonance frequency increases as confirmed by the calculations depicted in Fig. 3. Equal circularities lead to identical motions and do not alter the resonance frequencies. When an external magnetic field is applied, the equilibrium positions of the cores are deflected in dependence on their circularities. Equal circulations are equally deflected, opposite circulations are deflected in opposite directions. Then, the attractive force of the core dipoles is reduced due to their increased distance. According to the Thiele model the lateral deflection of the cores for the case of opposite circularities is 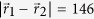 nm. The dependence of the deflections on the in-plane energies 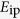 remains linear. Thus, the influence of the in-plane coupling is unaffected by the external magnetic field. The calculated spectra become identical to the case of pure in-plane coupling presented in Fig. 3. When the external magnetic field is increased to higher values, anharmonic contributions of the confining potential can occur^20^,^32^,^33^,^34^. These contributions could be used to slightly manipulate circularity and polarity dependent modes in the crystals.

Both the interaction between laterally coupled stacks and the circularity-dependent core-region coupling are small with respect to the coupling between in-plane dipoles of the stacked disks. Still, all presented interactions individually increase the number of non-degenerate circularity and polarity states in the crystal. The non-linear influence of the vortex cores can be reduced by small external magnetic fields and confirms the model of interacting out-of-plane dipoles.

We conclude that three-dimensional magnonic vortex crystals feature a wide range of excitable resonance frequencies. The spectra of the vortex arrangements are directly linked to the circularities and core polarizations of the vortices. The relative core polarization in a stack strongly influences the resonance frequency. The interaction of the cores yields an additional contribution to the shift of the resonance frequency which can be manipulated by external magnetic fields. The lateral interaction between neighbouring stacks is in the same order of magnitude and can be explained based on coupled Thiele equations.

## Additional Information

**How to cite this article**: Hänze, M. *et al*. Collective modes in three-dimensional magnonic vortex crystals. *Sci. Rep.* **6**, 22402; doi: 10.1038/srep22402 (2016).

## Supplementary Material

Supplementary Movie S1

Supplementary Video Legend

## Figures and Tables

**Figure 1:**
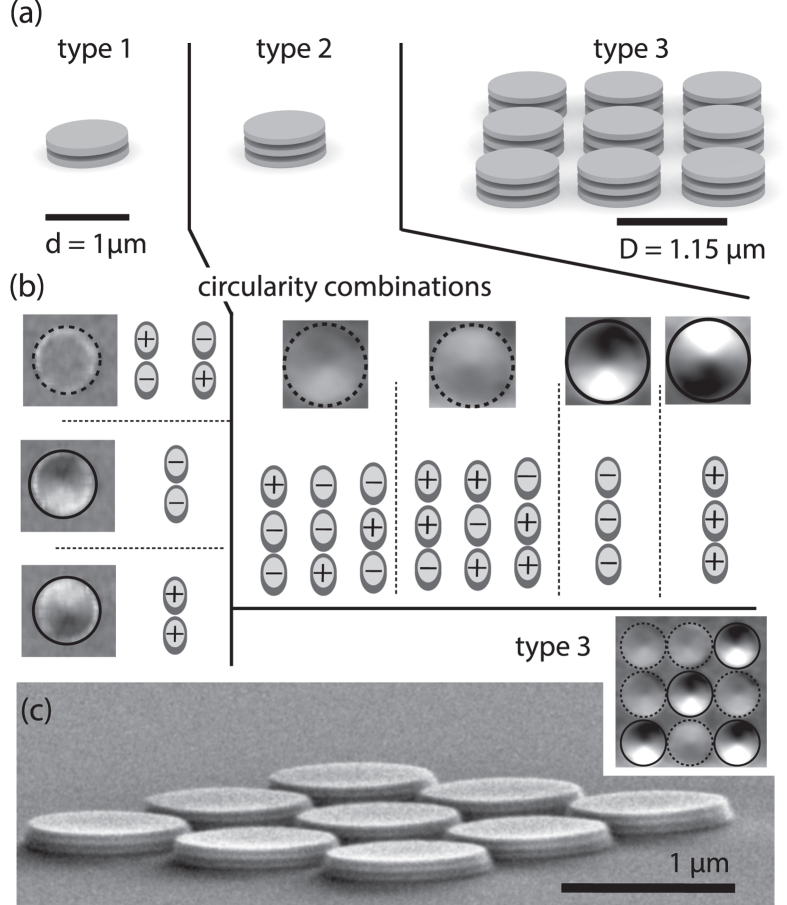
(**a**) Schematics of the investigated sample types: (1) isolated stack of two vortices, (2) isolated stack of three vortices, (3) 3 × 3 × 3 vortex crystal. (**b**) Scanning transmission X-ray micrographs of the in-plane components of the vortex stacks. The circularity combinations that lead to the depicted contrast are denoted by the +/− symbols. (**c**) Scanning electron micrograph of a vortex crystal with a scanning transmission X-ray micrograph as inset.

**Figure 2:**
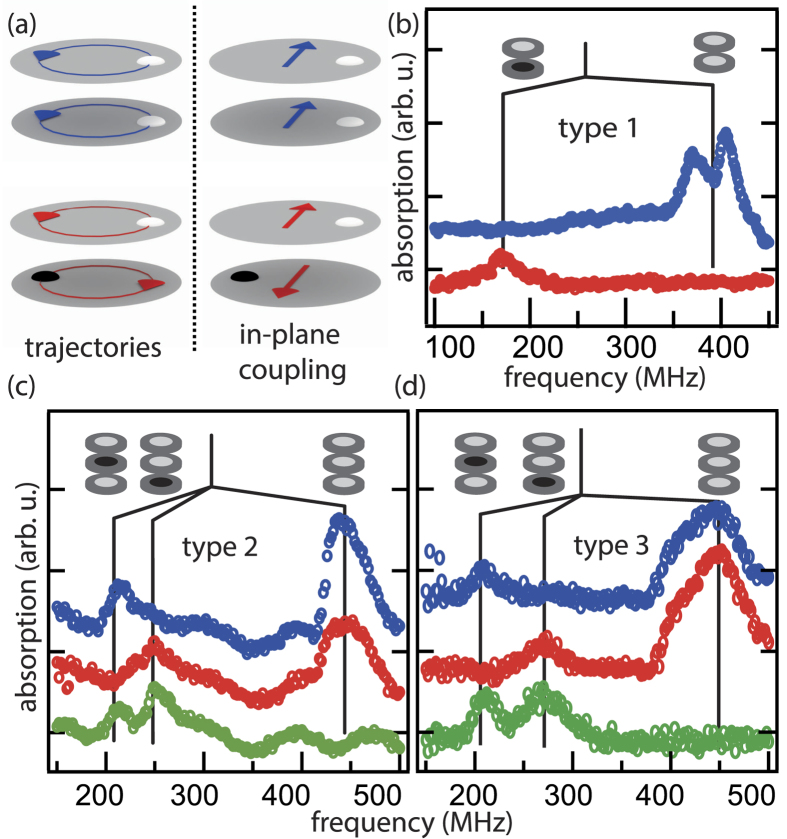
(**a**) Schematics of the vortex trajectories and the corresponding in-plane dipoles for the two possible relative polarizations (red *p*_1_*p*_2_ = −1, blue *p*_1_*p*_2_ = 1) of two stacked vortices with equal circularities. (**b**) Absorption spectra of an ensemble of sample type 1 for two different relative polarizations (red *p*_1_*p*_2_ = −1, blue *p*_1_*p*_2_ = 1). The vertical black lines indicate the resonance frequencies. Absorption spectra (**c**) of sample type 2 and (**d**) type 3. The color code indicates the frequency that is used to tune the polarity state (150 MHz/red, 300 MHz/blue, 450 MHz/green). The peaks correspond to the indicated relative polarizations within the stacks.

**Figure 3:**
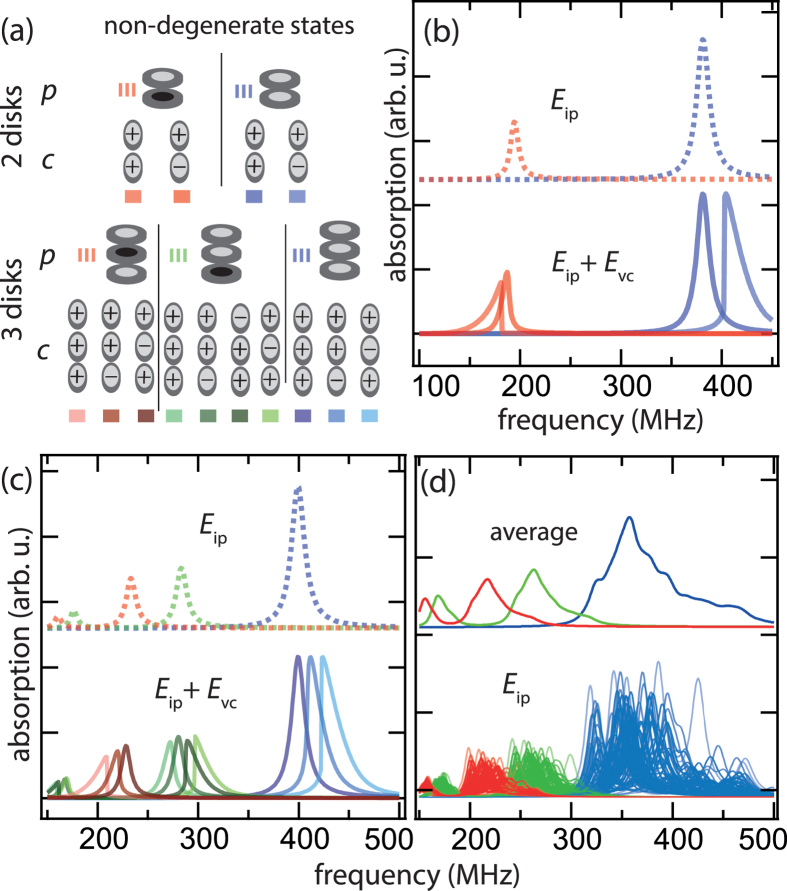
(**a**) Schematics of all non-degenerate states of different combinations of the polarities *p* and circularities *c* in stacks of two and three disks. (**b**) Calculated resonance spectra with (bottom) and without (top) considering the interaction of two stacked vortex cores. The spectra are depicted for all non-degenerate states. (**c**) Corresponding spectra for three vortices. (**d**) Resonance spectra of a 3 × 3 × 3 vortex crystal.

**Figure 4:**
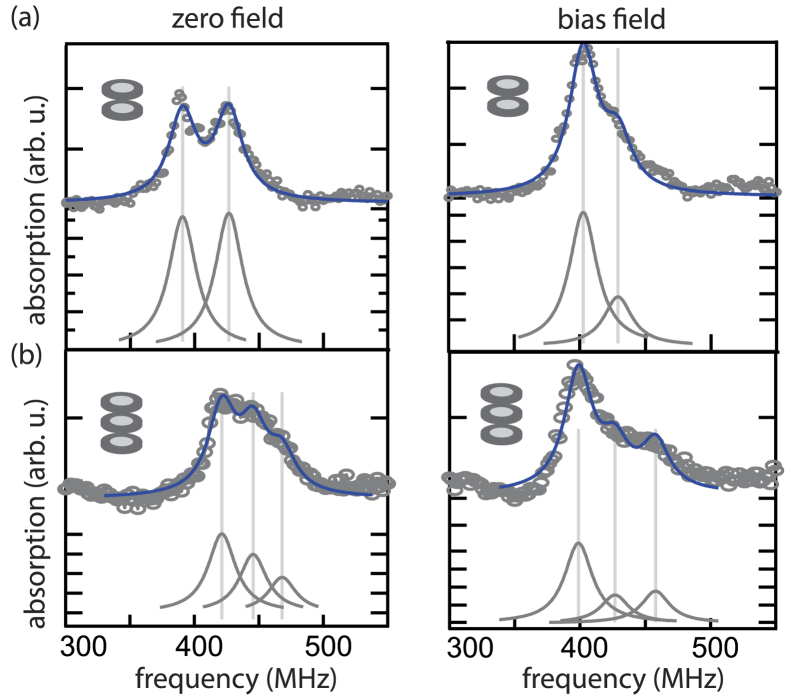
Resonance spectra of vortex stacks (a) of sample type 1 and (b) of type 2. The spectra are obtained for equal polarizations within the stacks. The spectra are shown without an external magnetic field and an in-plane field of 4 mT. Lorentzian curves are fitted to the data.
