# Supplementary material for: Collective modes in three-dimensional magnonic vortex crystals
Source: Sci Rep. 2016 Mar 2;6:22402. doi: 10.1038/srep22402 (PMC4773813; doi:10.1038/srep22402)
Supplement: Supplementary Video Legend [file srep22402-s2.pdf]

Collective modes in three-dimensional magnonic vortex crystals
Supplementary Information File

Max Hänze,[1,*] Christian F. Adolff,[1] Benedikt Schulte,[1]
Jan Möller,[1] Markus Weigand,[2] and Guido Meier[3,4,1]

[1]*Institut für Angewandte Physik und Zentrum für Mikrostrukturforschung, Universität Hamburg, 20355 Hamburg, Germany*

[2]*Max-Planck Institute for Intelligent Systems, Heisenbergstr. 3, 70569 Stuttgart, Germany*

[3]*Max-Planck Institute for the Structure and Dynamics of Matter, Luruper Chaussee 149, 22761 Hamburg, Germany*

[4]*The Hamburg Centre for Ultrafast Imaging, Luruper Chaussee 149, 22761 Hamburg, Germany*

I. VIDEO LEGEND - MOVIE 1

The supplementary movie 1 depicts the in-plane motions of a 3×3×3 vortex stack for two different polarization patterns. The patterns have been generated using state-formation frequencies of (left) 150 and (right) 450 MHz. The two patterns are excited by a sinusoidal unidirectional magnetic field at the indicated frequency. The crystal's vortices show strong gyrations at their resonance frequency. In the used in-plane setup the amplitude of the gyration is determined by the intensity of the black and white contrast as described in Ref. [1]. The resonance frequency is tuned by the polarization pattern of the vortices.

---

* max.haenze@physnet.uni-hamburg.de

[1] Behncke, C., Hänze, M., Adolff, C. F., Weigand, M. & Meier, G. Band structure engineering of two-dimensional magnonic vortex crystals, *Phys. Rev. B* **91**, 224417 (2015).
